# How Monitor Characteristics Affect Human Perception in Visual Computer Experiments: CRT vs. LCD Monitors in Millisecond Precise Timing Research

**DOI:** 10.1038/s41598-020-63853-4

**Published:** 2020-04-24

**Authors:** Michaela Rohr, Alexander Wagner

**Affiliations:** 1 Department of Psychology, Saarland University, Saarbruecken, Germany; 2 Department of Computer Science, Saarland University, Saarbruecken, Germany

**Keywords:** Psychology, Human behaviour

## Abstract

Liquid crystal display (LCD) monitors are nowadays standard in computerized visual presentation. However, when millisecond precise presentation is concerned, they have often yielded imprecise and unreliable presentation times, with substantial variation across specific models, making it difficult to know whether they can be used for precise vision experiments or not. The present paper intends to act as hands-on guide to set up an experiment requiring millisecond precise visual presentation with LCD monitors. It summarizes important characteristics relating to precise visual stimulus presentation, enabling researchers to transfer parameters reported for cathode ray tube (CRT) monitors to LCD monitors. More importantly, we provide empirical evidence from a preregistered study showing the suitability of LCD monitors for millisecond precise timing research. Using sequential testing, we conducted a masked number priming experiment using CRT and LCD monitors. Both monitor types yielded comparable results as indicated by Bayes factor favoring the null hypothesis of no difference between display types. More specifically, we found masked number priming under conditions of zero awareness with both types of monitor. Thus, the present study highlights the importance of hardware settings for empirical psychological research; inadequate settings might lead to more “noise” in results thereby concealing potentially existing effects.

## Introduction

With modern display technology becoming increasingly advanced, bulky cathode ray tube (CRT) monitors are (with few exceptions) no longer being produced. Instead, flat panel technologies have become the de-facto standard and among those, liquid crystal display (LCD) monitors are most prevalent. This technological change has also affected experimental research relying on computerized presentation of stimuli. Based on decades of experience with CRT monitors, their characteristics are well known and they have proven to provide reliable and precise stimulus presentation^1^. Early-generation LCD monitors, however, were less reliable and rather insufficient for precise stimulus presentation^1–3^. Thus, many researchers are still reluctant to equip their laboratories with the new technology^4^. Newer generation LCD monitors are assumed to have improved characteristics^5,6^, but the exact characteristics can vary substantially between models, making it difficult to know whether a specific model and specific settings result in the intended presentation duration and fidelity^2,3,7,8^. Elze and Tanner^8^, for example, highlighted differences in signal shape, luminance, response times (and therefore motion blur), and background illumination, not only between CRT and LCD monitors, but also between different models of the same monitor type, resulting in differences in both the physical and perceived duration of stimuli. Therefore, it is highly recommended to obtain measurements regarding actual technical equipment performance, rather than relying on specifications (e.g., frame rate) provided, especially when using short stimulus durations^3^. However, obtaining measurements might be difficult in practice due to the extensive knowledge and resources required. Moreover, while several articles have examined the technical properties of LCD monitors^1–3,5,6,9^, only few articles have investigated perceptual characteristics of CRT versus LCD monitors and how they relate to human performance^7,10,11^. Thus, even if researchers take technical differences into account, it remains unclear whether CRT and LCD monitors yield comparable perceptual effects, especially when it comes to short and millisecond-precise stimulus presentations (see, e.g^7^, for differences concerning display persistence).

The present paper summarizes the current knowledge base regarding important differences between CRT and LCD monitors; it aims to provide a hands-on guide for the setup of computer experiments using LCD monitors in a manner that yields reliable presentation times and CRT-comparable results. Additionally, we provide empirical evidence from a masked priming task and a prime-discrimination task, demonstrating that current-generation LCD monitors can be used for masked visual stimulus presentation.

## CRT and LCD Monitor Characteristics Relating to Visual Stimulus Presentation

First, we will provide a brief technical overview of functional principles as they relate to visual stimulus presentation. Detailed descriptions and parameter measurements are already available from the existing literature; however, our intention here is to equip readers with limited technical expertise with the necessary knowledge to set up computer experiments with LCD monitors. Thus, we keep our explanations relatively short and simplified.

### Cathode ray tubes (CRTs)

**Figure 1:**
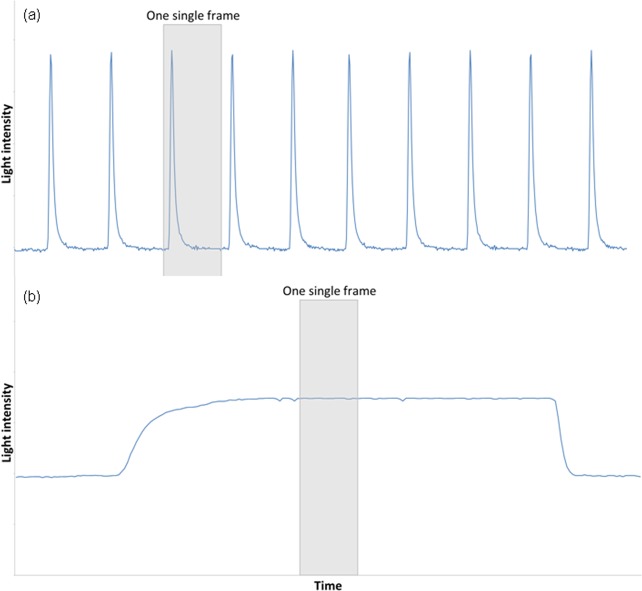
A pixel’s light intensity over time in (**a**) a CRT monitor (upper panel) and (**b**) an LCD monitor (lower panel).

CRT technology has been studied for decades^2,12,13^. CRTs consist of a bulky vacuum tube and a screen that contains a grid of phosphor-coated pixels; inside the tube, one or multiple electron beams perform a linewise scan across the pixel grid from left to right, and top to bottom, illuminating the phosphor particles. When the beam reaches the last pixel on the bottom right, it jumps back to the top left and restarts scanning (i.e., a new frame is drawn). This process typically repeats about 100 times per second (i.e., 100 Hz). As the electron beam can only hit one pixel at a time, the illusion of a continuously displayed image is both a result of the fast refresh rate (similar to the perception of continuous motion in films) and the fact that the phosphor illumination has a quick onset but is slow to subside. The upper panel in Fig. 1 (Fig. 1a) shows a pixel’s typical brightness profile over time for a static white CRT display.

### Liquid crystal display monitors (LCDs)

LCD monitors work differently: Each pixel consists of liquid crystal threads that can be twisted or arranged in parallel by an electrical current applied to them. This leads to a polarization effect that either allows or prevents light passing through. A white light source located behind this crystal array uniformly and constantly illuminates the array. To display a black pixel, the crystal threads are twisted by 90° such that no light will pass through. A white pixel is achieved by aligning the crystals such that maximum light is allowed to pass through, until a different, non-white color needs to be displayed (see the lower panel of Fig. 1 for an LCD pixel’s brightness over time). This is a static process, not a pulsed one as in CRTs.

#### Impact of technological differences on stimulus presentation

In theory, the difference in presentation methods, namely a strobing versus a static image, should be of no consequence if the light energy that falls onto the retina remains the same over the time period of one single frame. As the Talbot-Plateau law states^14^: “a flash sequence that is above the flicker-fusion threshold will be perceived as being equal in brightness to a steady stimulus when the average intensity of the former matches the intensity of the latter.” Indeed, the law holds in typical situations involving computer monitors: Photopic vision occurs when the environment is well-lit (approx. > 10 cd/m² ^15^), which is the case with typical computer monitors (e.g., our monitors were set to 110 cd/m²). The typical refresh rate of 100 Hz exceeds the 60–90 Hz needed to exceed the flicker-fusion threshold^16^. Likewise, Bloch’s law^2,17,18^ is assumed to hold for computerized presentation. It states that the detectability of shortly presented visual stimuli (i.e., until approx. 60–100 ms duration, see^17^) is determined by their energy, that is, the product of luminance and duration. As stated by [^19^, p.2444]: “For example, a light flash presented for 30 ms at a luminance of 80 cd/m^2^ is equally well detectable as a light flash presented for 60 ms at 40 cd/m^2^. This suggests that temporal integration can be easily described by energy summation”. Thus, in principle, LCD and CRT monitors should be able to yield comparable results.

However, due to the differences in technology, the visual signals produced by the two display types have different shapes (i.e., a different light energy-over-time-curve; see Fig. 1). Moreover, default luminance as well as visual-signal response times (in addition to other parameters, see below) differ between most CRT and LCD monitors^2,3,8^. Thus, the physical energy under default settings is likely to differ, making specification of presentation durations in terms of frames or milliseconds insufficient^3^. Rather, one needs to know the luminance over time (i.e., the exact shape of the light energy-over-time-curve) to estimate the physical energy. A specification in these terms is, however, usually not done in psychological experiments.

A further critical point is that early-generation LCDs typically had slow response times^1,6,8^, making them unsuitable for short stimulus presentations. Newer-generation LCDs, however, are equipped with technologies (i.e., dynamic capacitance compensation [DCC], which is often also termed “overdrive”) that compensate for this problem by speeding up gray-to-gray response times^6,9^. In simplified terms, this means that the monitor is equipped with an internal buffer that stores the frame currently requested by the computer’s graphic card while still displaying the previously-requested frame. This one-frame buffer time can be used for pre-calculations, allowing the panel’s capacitors to more rapidly flip the liquid crystals, results in faster transitions (but also an inevitable (constant) input lag of at least one frame^8^). Moreover, response times can vary depending on the exact luminance transitions^8^: A black-to-white transition is always slower than a dark-gray to light-gray transition. Therefore, researchers are advised to carefully evaluate whether a specific monitor can be used for millisecond-precise presentations or not. In principle, when taking into account brightness over time, and with DCC enabled for fast responses and appropriate luminance adjustments, CRT and LCD monitors should be able to yield comparable results^9^.

That said, some technological differences still remain, such as differences in display persistence^6^ and background illumination^2,3,8^. Further difficulties may arise from motion blur with LCDs and their greater dependency on viewing angle, which may cause unintended effects^8^. Thus, depending on the specific aims of their research, researchers are advised to take these properties into account. Our aim here was to examine whether LCD monitors can be used for fast and precise stimulus presentations; to this end, we designed a masked priming experiment, using central, static presentation of stimuli. Before conducting this experiment, we obtained technical measurements guided by the theoretical knowledge outlined above.

#### Configuration parameters and measurement results of the response characteristics of the employed CRT and LCD monitors

Table 1 reports the parameters we considered in setting up the CRT and LCD monitors. Certainly, most of them are commonly considered when setting up a computer experiment; nevertheless we deemed it important to mention them here explicitly, as their neglect might have unintended consequences. We used a 17” Fujitsu Siemens Scenicview P796-2 CRT color monitor previously used in several published studies including studies with masked presentation conditions^20–22^ and a thin film transistor monitor with twisted nematic (TN) panel. TN panels and “in-plane switching” (IPS) panels are the two currently dominant technologies. TN panels are typically superior in terms of input lag to IPS panels, but IPS panels often feature brighter colors, higher contrasts, and greater viewing-angle tolerance. Thus, researchers’ panel choice should be determined by the parameters they need to optimize for their research.

**Table 1:** Hard- and software settings to consider to achieve comparable results to CRT monitors with LCD monitors.

Feature	Description	Recommendation	Comment	Experiment setting
LCD panel type	IPS (in-plane switching): true-color and contrast less dependent on viewing angle, slower response time; TN (twisted nematic): fast response time, colors fade with non-optimal viewing angle.	Whenever timing is an issue: Use TN panels		In the present study, TN panels were used.
Native resolution, screen diagonal, and aspect ratio	With constant screen diagonal and aspect ratio: The higher the resolution, the smaller objects and stimuli that are measured in pixels appear on the screen.	To achieve results as close as possible to a CRT experiment, calculate the size (e.g., in mm) of one native pixel and resize the stimuli if necessary, so that the real size (in mm) on the CRT corresponds to the real size on the LCD.	Take the aspect ratio into account to avoid distortions like they would appear when a resolution with an aspect ratio of 4:3 (e.g., 1024 * 768) is applied to a monitor with a native aspect ratio of 16:9 (e.g., native resolution of 1920 * 1080). If you need to do the latter, consider letterboxing.	In the present study, CRT resolution was 1024 * 768 (visible area 324 * 243 mm, aspect ratio 4:3), diagonal 17”, dimensions of 1 pixel: 0.316 * 0.316 mm. LCD resolution was 1024 * 768 (visible area 531 * 299 mm, aspect ratio 16:9, dimensions of 1 pixel (letterboxed to 4:3) was 0.389 * 0.389 mm). LCD stimulus size thus needed to be enlarged by a factor of 1.23. Stimuli were adjusted to match sizes.
Monitor brightness (as can be set in the monitor’s user menu)	Provides the same amount of radiated energy in a single frame compared to CRTs.	Measure the brightness of a used (and warmed up) experimental CRT with a luminance meter with both a completely black and a completely white screen. Try to match both values with the LCD.	When an exact match is not possible, try to adjust the monitor’s contrast setting accordingly (i.e., usually downregulate the LCD).	In the present study, CRT settings used an on-screen-display brightness setting of 100%; LCDs were set to 9%.
Refresh rate	Multiple complex effects are dependent on the choice of the correct refresh rate, particularly the multiples of the presentation time of a single frame.	Choose the refresh rate to match your CRT or, when designing a new experiment, to match your desired stimulus presentation times as closely as possible.	Example: Stimulus presentation 30 ms; typical refresh rates are 60, 70, 100, 120, 144 Hz. Possible choices are two frames of 60 Hz = 2 * (1/60) = ca. 33 (ms). A better choice would be three frames of 100 Hz = 3 * (1/100) = 30 (ms).	The experiment in the present study used a refresh rate of 100 Hz with presentation times consisting of multiples of 10 ms.
DCC (dynamic capacitance compensation)	Faster gray-to-gray response times at the cost of a constant delay of approx. one frame.	Turn on when possible.	Signals tend to slightly overshoot a few percent brighter than intended, typically for approx. 1 ms.	

We tested various monitor user settings, refresh rates, resolutions and luminance settings (see materials available at https://osf.io/g842s/) with regard to the emitted light energy–over-time-curve and therefore response characteristics (i.e., onset and offset of full screen and centrally presented stimuli). Measurements were conducted with a photodiode setup, using both an oscilloscope (model “Agilent MSOX 3012 A”) and a self-developed microcontroller setup as measurement devices. Stimuli were black and white squares.

Our measurements revealed several interesting characteristics: First, luminance of the LCD monitor at default setting (i.e., maximum brightness) exceeded the CRT luminance at a ratio of 3.25:1. However, comparable average luminance can be (and was) achieved by downregulating the LCD monitor (the older CRT technology emits less energy even at maximum settings, see Table 2), without participants perceiving it as unnaturally dark. If one plans to upgrade from CRT to LCD monitors in an experimental laboratory, we therefore recommend measuring the CRT monitors’ brightness levels and matching them in the new LCD monitors’ user setup, if comparability with the old setup is needed. This will minimize hardware-dependent variability, thus contributing to better replicability. Please note that a brightness adaption is not a necessary precondition when employing LCD monitors; researchers should simply be aware that the brightness level can have an influence onto the resulting effects, especially in time-critical experiments with short and/or masked presentation. Thus, we recommend the adaptation for time-critical experiments in which researchers orient on existing empirical evidence gathered with CRT monitors. Furthermore, gray-to-gray response times varied slightly depending on the employed brightness levels^8^, and as intended by the manufacturer, changes in gray-to-gray response times were consistently faster with DCC enabled than without DCC. We also compared the oscilloscope measurements with the measurements of a luminance meter (model “Gossen Mavo-Monitor USB”), which allows for more time- and resource-efficient measurements. Indeed, the luminance meter yielded comparable results (see Table 2), so we suggest that researchers can rely on this more efficient method as an approximation.

**Table 2:** Average light energy measured in specific settings.

Monitor type	Brightness (in %)	Stimulus color	cd/m²	Volt
CRT	100	white	108	1.34
CRT	100	black	4.58	0.99
LCD	100	white	358	2.19
LCD	20	white	139	1.46
LCD	9	white	110	1.35
LCD	9	black	0.27	0.95

*Note*. Brightness refers to monitor menu settings, cd/m² was measured with the luminance meter and also calculated from the measured voltage (i.e., via oscilloscope). The voltage function matches the values measured with the luminance meters almost perfectly.

For the empirical comparison of human performance with CRT and LCD monitors, we relied on these results and set the monitor settings accordingly (see Method section below).

#### Masked number priming using CRT and LCD monitors

Participants were administered a masked number priming task and a subsequent forced-choice prime discrimination task using both a CRT and an LCD monitor. In this well-established paradigm^23–25^, participants classify one-digit target numbers (e.g., as smaller or greater than 5); targets are preceded by briefly presented, masked one-digit primes, which facilitate target classification if they are congruent with the target (i.e., both prime and target smaller or greater than 5) but hinder classification if they are incongruent. The resulting RT difference between congruent and incongruent trials is known as the masked priming effect. It has been shown that even primes that are not included in the target set and are thus never visibly shown can elicit priming effects, providing evidence for processing at a semantic level^24,25^.

Of central interest was the question whether both monitors would yield comparable masked priming effects. Monitors were set according to the parameters described in the previous section (see also Method section below). In order to obtain conclusive evidence, we decided for sequential hypothesis testing using Bayes factors^26^. The study was preregistered on the Open Science Framework (OSF); all materials and data are available at https://osf.io/g842s/.

## Method

### Participants

As we aimed to find evidence for or against monitor type differences in priming, we applied sequential hypothesis testing with Bayes factors (BF), which allow quantification of evidence both for and against a null hypothesis^26^. After an initial sample of *n* = 24 was collected (see preregistration), we continued data collection until the preregistered BF (with JSZ prior *r* = 1) was reached. Specifically, data collection was stopped after the BF reached either (a) BF_01_ > 6 in favor of the null hypothesis of no difference in priming effects for CRT and LCD monitors, or (b) BF_10_ > 6 in favor of the alternative hypothesis that there is a difference between CRT and LCD monitors. We computed the BF after each day of data collection, and the critical BF was reached after testing 68 participants.

Participants were non-psychology students from Saarland University (40 females, 25 males; age *Md* = 25 years, range: 18–36), who were compensated with €8. Participants gave written informed consent before the study, and were free to withdraw from the study at any point in time. Anonymity of data was ensured, and treatment of subjects was in accordance with the Declaration of Helsinki. According to the guidelines of the German Research Association (Deutsche Forschungsgemeinschaft; DFG), no ethical approval was needed for this study (http://www.dfg.de/foerderung/faq/geistes_sozialwissenschaften/index.html) because it did not pose any threats or risks to the participants and participants were fully informed about the objectives of the study. The chairman of the Ethics Committee of the Faculty of Empirical Social Sciences of Saarland University confirmed that ethical approval was not needed for this study.

All participants had normal or corrected-to-normal vision. Data from one participant were excluded because of an outlying error rate, as specified in the preregistration (i.e., using an outlier criterion of 27.5% computed based on the initial sample of *n* = 24); one participant erroneously took part in the experiment twice; another participant’s data were lost due to experimenter error. Thus, final sample size for analysis was *N* = 65. All data (including the excluded data files) are available at https://osf.io/g842s/.

### Design

The experiment was a replication of Kunde *et al*. 2003, Exp.1^25^ with some minor exceptions (i.e., 100 Hz refresh rate instead of 70 Hz; black background instead of a dark-grey background in Kunde *et al*.’s experiment). Participants’ task was to classify one-digit target numbers as smaller or greater than 5. Preceding the targets, sandwich-masked number primes were presented. The basic design of the priming task was a 2 (prime: smaller/greater than 5) × 2 (target: smaller/greater than 5) × 2 (monitor type: CRT vs. LCD) within-participants design. Following Kunde *et al*.^25^, the design was transformed into a 2 (priming condition: congruent vs. incongruent) × 2 (monitor type: CRT vs. LCD) design for analysis. The monitor factor was manipulated block-wise (i.e., participants switched once between monitors for both the priming task and the prime-recognition task), with monitor order counterbalanced. As in Kunde *et al*.^25^, Exp.1, both prime and target numbers were presented as either Arabic numerals or number words. Also, the target set formed only a subset of the prime set. Thus, match of notation (Arabic vs. verbal; i.e., prime and target have the same or different format), and prime novelty (i.e., prime is part of the target set or not) were additional factors. Kunde *et al*. did not find an impact of these factors on the congruency effect; they were, however, included for replication purposes (As a side effect, Kunde *et al*. found an interaction of notation match x congruency x prime novelty indicating small differences in masking efficiency due to greater/smaller overlap in prime-target shape; we also found such an effect, see below).

### Materials

We used exactly the same materials as Kunde *et al*., Exp. 1^25^. The prime set comprised the Arabic numbers “1” to “9” (except “5”) and the corresponding German number words in capital letters (e.g., “EINS”). The target set comprised numbers 1, 4, 6, and 9 (and the corresponding number words). Masks were strings of six upper-case consonants, randomly drawn for each presentation. All stimuli were displayed with a letter size of 0.5 × 0.5 cm and approx. 4° of visual angle for the longest stimulus (“SIEBEN”) at a monitor distance of 60 cm (i.e., they were presented in a monospaced font with a 15 pt font size for the CRT monitor and a 12.5 pt font size for the LCD monitor; stimuli needed to be size-adjusted due to variations in display size and aspect ratio).

### Apparatus

We used two 17” Fujitsu Siemens Scenicview P796-2 CRT color monitors and two 24” ViewSonic VG2401mh TFT monitors, all set to a resolution of 1024 × 768 pixels, and a refresh rate of 100 Hz . Luminance on both monitors was set to 110 cd/m² (using the luminance meter model “Gossen Mavo-Monitor USB”). The room was completely dark (i.e., measured background luminance was less than 0.5 cd/m²). Stimulus presentation and measurement of response latencies were controlled by E-Prime version 2.0 run on a DELL PRECISION T1600 computer. Participants gave their responses with a standard QWERTZ keyboard connected via PS/2. They sat at a distance of approx. 60 cm to the monitor. Distance to the monitor and viewing angle were measured at the beginning of each task (i.e., with each monitor change) and visually monitored by the experimenter in regular intervals.

### Procedure

Up to two individuals participated concurrently, separated by partition walls. Participants were randomly assigned to a monitor order (CRT or LCD first), and switched monitors twice, that is, they first completed the priming task on monitor 1, then the same priming task on monitor 2 [or vice versa]. Afterwards, they switched again to monitor 1 for the prime discrimination task, and then executed the prime discrimination task again at monitor 2 [or vice versa]).

#### Priming task procedure

**Figure 2:**
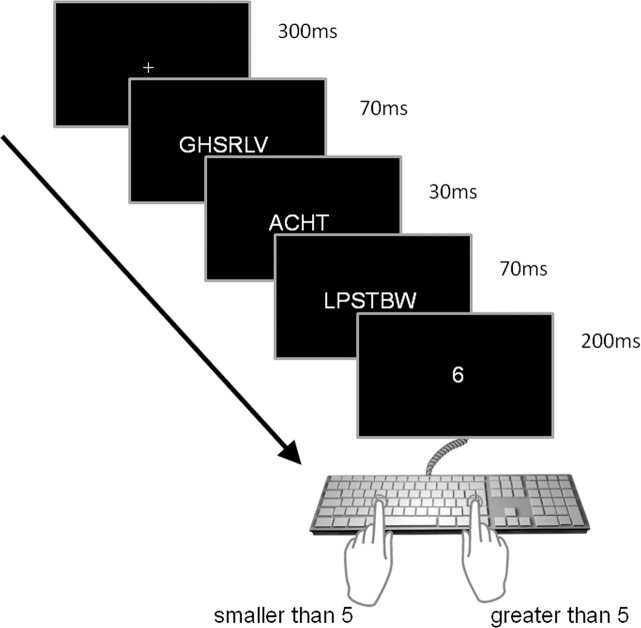
Example of a trial in the priming and prime-recognition task.

The trial sequence was as follows: First, a fixation cross was displayed for 30 frames (i.e., 300 ms), followed by a pre-mask presented for seven frames (i.e., 70 ms), the prime presented for three frames (i.e. 30 ms), and a post-mask for seven frames (i.e., 70 ms; SOA = 100 ms). The post mask was immediately followed by the target, which was presented for 20 frames (i.e., 200 ms), followed by a blank (black) screen for 200 frames (i.e., 2,000 ms), which signaled the response deadline. Response keys were the ‘f’ and ‘j’ keys on a standard German QWERTZ keyboard, marked with blue stickers. If a response was given, immediate feedback (“Richtig!”/“Falsch!”; i.e., “Correct!”/“Wrong!”) was provided. After an inter-trial-interval of 800 ms, the next trial started. Figure 2 depicts an example trial.

At the beginning of the experiment, participants were informed that the experiment was investigating the differences between CRT and LCD computer monitors and that they were therefore asked to work on a simple number-categorization task using different monitors. They were instructed to categorize the presented numbers as quickly and accurately as possible. They were not informed about the primes. To familiarize participants with the procedure, they first received a practice block of 32 trials. The actual experiment consisted of five blocks of 128 trials each. After each block, participants were free to take a short break.

#### Prime discrimination task procedure

In the direct test of prime discrimination, which is typically included in masked priming experiments^23–25^, participants worked through two blocks of 128 trials each. All task parameters were identical to the priming task, except that participants were informed about the primes and instructed to categorize them instead of the targets. Moreover, no response feedback was provided and participants were not under time pressure to respond, that is, the blank screen was shown until a response was made.

## Results

Mean response latency for correctly categorized targets was the dependent variable of interest. Data preparation and analysis were done as preregistered, that is, trials with reaction times below 150 ms or more than 3 interquartile ranges above the third quartile or below the first quartile of the individual distribution were discarded (1.06% of all trials), as were trials with incorrect responses (*M* = 6.48%, *SD* = 4.42%, range from 1.09% to 20.39%). Table 3 shows mean reaction times and error rates across conditions.

**Table 3:** Mean reaction times in milliseconds and (percentage errors in parentheses) across monitor type, notation match, and prime novelty conditions.

	Monitor-Type							
	CRT				LCD			
	congruent		incongruent		congruent		incongruent	
Prime novelty	match	non-match	match	non-match	match	non-match	match	non-match
practiced	483 (6)	484 (6)	487 (7)	485 (7)	511 (7)	504 (7)	510 (7)	510 (7)
unpracticed	485 (6)	483 (6)	485 (6)	484 (6)	509 (6)	508 (6)	511 (7)	511 (6)

In the following, we present the Bayes factors based on sequential hypothesis testing as preregistered (computed with JASP, version 0.10.2), alongside results of conventional null-hypothesis significance testing (NHST) on the final sample (conducted with SPSS, version 26) to allow comparison with the original results of Kunde *et al*.^25^. The final sample of *N* = 65 provided sufficient power to detect effects of *d*_*Z*_ = 0.35 (i.e., between small and medium size according to^27^).

### Priming effects

As our central hypothesis regarded the (lack of) priming differences between monitor types, we first present the Bayesian analysis assessing the interaction of priming condition (congruent, incongruent) and monitor type (CRT vs. LCD).

#### Sequential hypothesis testing

**Figure 3:**
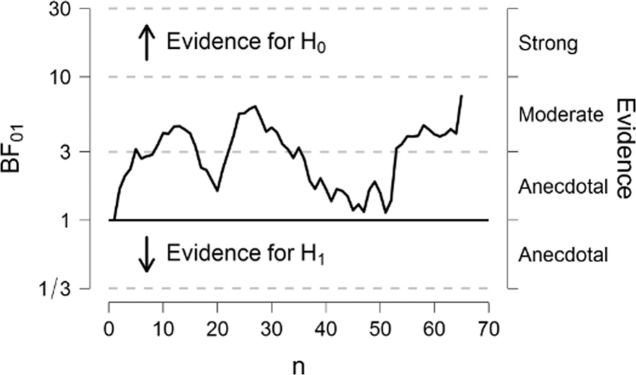
Development of the Bayes Factor BF01 in favor of the null hypothesis for the priming condition × monitor type interaction term across the course of the experiment.

The final BF01 for the interaction of priming condition and monitor type was BF01 = 7.47; this means that the data are approx. 7.5 times more likely under the null, and thus represents moderately strong evidence for the hypothesis that the two monitor types produced equivalent masked priming effects. The evolution of the BF01 can be seen in Fig. 3. Overall there was also strong evidence for the presence of a small priming effect (*M* = 1.92 ms, *SD* = 4.15 ms, *d*_*Z*_ = 0.46) with BF10 = 46.62.

#### NHST analyses

The 2 (priming condition: congruent vs. incongruent) × 2 (monitor type: CRT vs. LCD) × 2 (notation match: match vs. non-match) × 2 (prime novelty: practiced vs. unpracticed primes) repeated measures ANOVA yielded significant main effects of priming condition, *F*(1,64) = 13.82, *p* < 0.001, ${\eta }_{p}^{2}$ = 0.178 (*d*z = 0.46), monitor type, *F*(1,64) = 99.11, *p* < 0.001, ${\eta }_{p}^{2}$ = 0.608 (*d*z = 1.23), and notation match, *F*(1,64) = 5.33, *p* = 0.024, ${\eta }_{p}^{2}$ = 0.077 (*d*z = 0.29). Furthermore, a significant three-way-interaction of priming condition × monitor type × notation match emerged, *F*(1,64) = 7.00, *p* = 0.010, ${\eta }_{p}^{2}$ = 0.099 (*d*z = 0.33). No further results were significant (for the sake of interest: priming condition × prime novelty, *F*(1,64) = 2.55, *p* = 0.115, ${\eta }_{p}^{2}$ = 0.038 (*d*z = 0.20); priming condition × notation match, *F*(1,64) = 2.16, *p* = 0.147, ${\eta }_{p}^{2}$ = 0.033 (*d*z = 0.18); priming condition × monitor type × notation match × prime novelty, *F*(1,64) = 2.77, *p* = 0.101, ${\eta }_{p}^{2}$ = 0.042(*d*z = 0.21)). We also checked for a possible effect of monitor order; no effects emerged. Please note that the main effect of monitor largely reflects the DCC input lag (see Introduction), that is, the recorded response times are larger for the LCD monitor, because the internally recorded stimulus onset time is earlier than it actual was due to the input lag.

We followed up the significant three-way interaction with separate ANOVAs for each monitor type. The repeated measures ANOVA for the LCD monitor yielded a significant priming condition × notation match interaction, *F*(1,64) = 8.16, *p* = 0.006, ${\eta }_{p}^{2}$ = 0.113 (*d*z = 0.35), while the interaction was not significant for the CRT monitor, *F*(1,64) = 0.58, *p* = 0.45, ${\eta }_{p}^{2}$ = 0.009 (*d*z = 0.09). In the LCD monitor analysis, prime-target combinations with non-matching format yielded a congruency effect, *t*(64) = 4.54, *p* < 0.001, *d*_*Z*_ = 0.56, while matching prime-target combinations did not yield a congruency effect, *t*(64) = 0.22, *p* = 0.83, *d*_*Z*_ = 0.03. It is likely that differences in masking efficiency were responsible for this finding (i.e., stimuli matching in format mask each other better), as Kunde *et al*.^25^ found a similar effect with a CRT monitor. We will further elaborate on this in the Discussion.

### Prime discrimination

The signal detection index *d’* served as the dependent variable in the prime-recognition task. In a first analysis, *d’* was tested against zero with a repeated-measures MANOVA, with monitor type as a within-participants factor. The constant test of this MANOVA was not significant, *F*(1,64) = 0.01, *p* = 0.94, ${\eta }_{p}^{2}$ = 0.000 (*d*z = 0.01), indicating overall chance performance. The main effect of monitor type was also not significant, *F*(1,64) = 0.59, *p* = 0.45, ${\eta }_{p}^{2}$ = 0.009 (*d*z = 0.10), indicating zero awareness with both monitor types (*d’*CRT = 0.004; *d’*LCD = −0.005).

A repeated measures ANOVA with notation (Arabic vs. verbal), prime novelty, and monitor type as within-participants factors yielded a notation × prime novelty interaction as the sole significant effect, *F*(1,64) = 6.20, *p* = 0.015, ${\eta }_{p}^{2}$ = 0.088 (*d*z = 0.31). Practiced digits were recognized better than unpracticed digits (*d’*prac_digits = 0.021; *d’*unprac_digits = −0.009), *t*(64) = 1.97, *p* = 0.05, *d*_*Z*_ = 0.24, while there was no such effect for number words, *t*(64) = 1.80, *p* = 0.08, *d*_*Z*_ = 0.22 (*d’*prac_words = −0.019; *d’*unprac_words = 0.006). Indeed, recognition was different from chance performance for practiced digits, *t*(64) = 2.16, *p* = 0.034, *d*_*Z*_ = 0.25, but not for any other item type, *ts* < 1.

## Discussion

The present paper contributes in important ways to empirical investigations of effects that necessitate millisecond-precise timing, such as the masked priming effects inspected in this paper. We laid out important differences between CRT and LCD technology, and provided guidelines on how to configure a current-generation LCD monitor to achieve results comparable to those obtained with a CRT monitor. Thus, our paper may help researchers establish adequate conditions to conduct such experiments with the precision needed, using state-of-the-art technology. Empirically, we demonstrated that experiments requiring precise timing—in this case a masked priming experiment—can yield comparable effects using CRT and LCD monitors. Specifically, we found comparable masked number priming effects using CRT and LCD monitors under conditions of zero prime awareness (with the exception of the practiced digits condition), as assessed with a separate forced-choice prime discrimination task. Thus, we replicated and extended the findings of Kunde *et al*.^25^ using a more contemporary technology. We deem this important not only because it provides further evidence for non-conscious processing, which is sometimes debated^28^, but also because a variety of effects in psychology seem difficult to replicate^29^. Our study can contribute to this debate by highlighting the perhaps under-appreciated issue that small differences in hardware settings or software might also have an impact on observed effects. We will first again summarize the most important issues concerning hardware setup and settings, and will then further discuss our empirical findings, especially as they relate to those technological issues.

First of all, the present paper shows that current-generation LCD monitors can be used for millisecond-precise presentation, even under masked presentation conditions. To this end, we used a twisted nematic (TN) panel, enabled DCC, used high-contrast stimuli, and adjusted the luminance of the LCD screen to yield a result comparable to a CRT monitor, given a predetermined stimulus presentation time. As we outlined extensively in the theoretical introduction, and as already stated by several other authors^2,3,8^, our results can certainly not be generalized to every LCD monitor and setting, given the parameter variability of different monitor models. However, we think that considering the differences in the shape of the energy curve, in (default) luminance, and in the specific characteristics of LCD panels (i.e., slower response times that can be compensated by DCC) can help researchers achieve precise and reliable presentation. The practical information we provided equips researchers with sufficient knowledge to check these parameters, which are often neglected in psychological research. Our measurements showed that a time- and resource-efficient luminance meter can be used for these purposes.

Regarding our empirical findings, we found, as hypothesized, significant and comparable masked number priming effects using both CRT and LCD monitors under conditions which yielded (for all except one condition) zero awareness in a subsequent forced-choice prime discrimination task. The Bayes factor evaluating a difference in priming effects between CRT and LCD monitors—the preregistered main hypothesis that provided the basis for data sampling—indicated strong evidence for the null hypothesis. Thus, the present results show that LCD monitors are suited for research requiring millisecond-precise timing, and that such research can yield comparable results to those obtained with a CRT monitor if luminance is matched and settings are chosen appropriately.

The detailed NHST analyses showed, as in Kunde *et al*.^25^, that priming effects were generally not moderated by prime novelty or notation match. However, we note that a significant priming condition by monitor type by notation match interaction emerged: Matching prime-target combinations yielded a weaker priming effect compared to non-matching prime-target combinations when using an LCD monitor, while no such result was found with the CRT monitor. Interestingly, Kunde *et al*.^25^ found a similar result with a CRT screen, albeit only for practiced primes, and argued that this effect was due to stronger backward masking by stimuli of the same format. We think that this explanation also applies to the present context. But why, then, did it not emerge when using a CRT monitor? We cannot provide a satisfactory answer to this question, although we speculate that the effect’s occurrence may relate to variance in perception. As the direct test revealed that there was some slight variance in perception between the LCD and the CRT monitors, effects of masking might also underlie these variations. However, we do not think that the effect reflects a real difference between CRT and LCD monitors, especially given the fact that there was no overall difference between the monitor types. Rather, some variation due to individual differences (or fluctuation across the experiment) in attention, concentration, motivation, and other factors might cause some variance in the effects. Certainly, the question of how different technologies might impact the mechanisms of masking^30^ is an interesting question, which might be followed up by future research. Of further note, we obtained priming effects ranging from 1–6 ms, while Kunde *et al*.^25^ obtained priming effects in the range of 8–17 ms. We do not think that this difference in size is related to the priming effects per se, but rather that differences in the technological equipment, computer or task settings might be responsible for the difference: First, degree of masking might have been – for unknown reasons (i.e. interindividual variability, variability in hardware, software etc.) - slightly different in our study compared to Kunde *et al*.’^25^ study. The signal detection indices in our study were lower than in their study (i.e., *d*’ = 0.29 in Exp.1 of Kunde *et al*.^25^). Such a difference in masking can easily translate into differences in priming effects. Second, Kunde *et al*.^25^ and we used different technological equipment: different screens, different computers, different keyboards. It is known that all these components can cause variation in measurement (see, e.g.^31^,). Thus, although we tried to measure as precisely as possible, it might be that data registration or transmission from the keyboard was associated with more variance than in Kunde *et al*.’s^25^ experiments. These possibilities show again how important technology, hardware and software settings are for computerized presentation in general, and millisecond precise presentation in particular. Slight differences can already lead to slightly different effects. Such small masked priming effects are, however, not untypical (see, e.g.^32^).

To summarize, the present empirical results showed that LCD monitors can be used for research requiring millisecond-precise timing, which can yield results that are comparable to those obtained from research conducted with CRT monitors, if settings are chosen appropriately. Our study thus highlights the importance of considering the effects of technological setup on empirical research. We hope that researchers in the field can use the recommendations we provided to achieve high precision in visual stimulus presentation.

## Data Availability

The empirical experiment was preregistered. The preregistration, as well as all data, analysis scripts, and experimental materials are available at (https://osf.io/g842s/).
